# Influence of Autochthonous Lactic Acid Bacteria Cultures on the Microbiota and Biogenic Amine Production in Medium-Ripened Artisan Goat Cheese

**DOI:** 10.3390/foods14091561

**Published:** 2025-04-29

**Authors:** Julia Mariano Caju de Oliveira, Adriane Elisabete Costa Antunes, Gustavo Felipe Correia Sales, Camila Neves Meireles Costa, Angela Matilde da Silva Alves, Kaíque Yago Gervazio de Lima, Celso José Bruno de Oliveira, Antônio Silvio do Egito, Karina Maria Olbrich dos Santos, Evandro Leite de Souza, Maria Teresa Bertoldo Pacheco, Maria Elieidy Gomes de Oliveira

**Affiliations:** 1 Department of Nutrition, Health Sciences Center, Federal University of Paraíba, João Pessoa 58051-900, Brazil; juliacaju0@gmail.com (J.M.C.d.O.); camilanevesmc@gmail.com (C.N.M.C.); els@academico.ufpb.br (E.L.d.S.); 2 School of Applied Sciences, State University of Campinas (FCA/UNICAMP), Limeira 13484-350, Brazil; adriane@unicamp.br; 3 Department of Animal Science, Federal University of Paraíba, Areia 58397-000, Brazil; gfcsales9@gmail.com (G.F.C.S.); celso.bruno.oliveira@gmail.com (C.J.B.d.O.); 4 Department of Food and Nutrition, Faculty of Food Engineering, State University of Campinas, Campinas 13083-862, Brazil; a235315@dac.unicamp.br; 5 Natural Products and Bioactive Synthetics, Center of Health Sciences, Federal University of Paraíba, João Pessoa 58051-900, Brazil; kaique.gervazio@gmail.com; 6 Embrapa Goats and Sheep, Northeast Regional Center, Campina Grande 58428-095, Brazil; antoniosilvio.egito@embrapa.br; 7 Brazilian Agricultural Research Corporation (Embrapa), Rio de Janeiro 23020-470, Brazil; karina.dos-santos@embrapa.br; 8 Center of Science and Food Quality, Institute of Food Technology, Campinas 13070-178, Brazil; mtb@ital.sp.gov.br

**Keywords:** autochthonous probiotic cultures, goat coalho cheese, microbiota modulation, metataxonomic sequencing, biogenic amines

## Abstract

This study evaluated the effects of adding the autochthonous cultures *Limosilactobacillus mucosae* CNPC007 (LM) and *Lactiplantibacillus plantarum* CNPC003 (LP), originally isolated from goat milk and goat cheese, respectively, on microbiological safety, microbiota composition (analyzed through 16S rRNA gene metataxonomic sequencing), and biogenic amine (BA) production in artisanal goat (*coalho*) cheese made from raw or pasteurized milk during 60 days of ripening at 10 °C. Six types of cheese were produced, varying in milk treatment (raw or pasteurized) and the presence or absence of LP or LM cultures. Adding either LP or LM significantly modulated the microbiota, favoring *Streptococcus* dominance and reducing overall bacterial diversity compared to non-inoculated cheeses. Raw milk cheeses with added autochthonous cultures exhibited a microbial profile like pasteurized cheeses, suggesting a homogenizing effect on the microbiome. Both cultures effectively reduced microbial load in raw milk cheeses after 20 days, reaching levels comparable to pasteurized cheeses by the end of ripening. Although BA concentrations increased over time, all samples remained within safe limits. Cheeses with LP addition exhibited lower BA levels, suggesting a modulating effect on their biosynthesis. Histamine concentrations were higher in raw milk cheeses with added cultures but remained well below hazardous levels. These findings suggest that incorporating either LP or LM strains is a promising strategy for enhancing the microbial safety and standardization of artisanal goat cheese while preserving its traditional characteristics.

## 1. Introduction

Coalho cheese is a traditional dairy product native to the Northeastern region of Brazil, where it has been produced and consumed for over 150 years. It holds significant economic and cultural value in this region, supporting local economies and small-scale dairy producers. It is characterized as a semi-hard cheese with a white color and salty and slightly acidic taste and can be made from either cooked or semi-cooked curd, consumed fresh or aged, and is one of the most consumed and commercialized cheeses in the country [1,2]. Although typically produced with cow’s milk, it can also be made using goat milk, which has been shown to have significant nutritional and functional potential, especially due to its relatively high protein content and improved fatty acid profile, serving as source of bioactive peptides and conjugated linoleic acid [2,3,4,5]. Usually, coalho cheeses produced with goat milk are often made in artisanal manners [6].

Artisanal cheeses are handmade using traditional techniques, often with raw milk, and produced on small-scale farms or dairies, closely tied to specific regions or cultures [7,8,9]. Consumer interest in artisanal cheeses is growing due to the more intense and complex flavors, superior texture, and aroma compared to standardized industrial cheeses. These characteristics are primarily attributed to the richer and more diverse microbiota in artisanal cheeses [8,9]. The poorer microbial diversity in industrial cheeses is a direct consequence of milk pasteurization, a process aimed at ensuring microbiological safety [7,8].

The concern around preserving the traditional manufacturing of artisanal cheeses is a worldwide matter, as seen in studies regarding the Protected Designation of Origin (PDO) cheeses, such as Silter cheese from Italy and Galotyri from Greece [10,11]. Alternatives such as extended ripening periods and the addition of lactic acid bacteria (LAB) strains, such as probiotics, are commonly employed to maintain the microbiological safety of raw-milk cheeses without compromising their sensory quality. Ripening is a complex process during which cheese develops its characteristic flavor, aroma, and texture through enzymatic reactions involving lactose fermentation, proteolysis, amino acid catabolism, and lipolysis, all of which are closely related to the presence of indigenous bacteria capable of performing physiological and biochemical functions of technological interest [9,10]. Studies by Silvetti et al. [10] and Tsanasidou et al. [11] have implemented the use of native strains in PDO cheeses aiming to optimize the fermentative process, preserving the biodiversity and authenticity of the product, while avoiding sensory defects due to microbial and compositional changes in raw milk.

Despite the desirable qualities, the ripening process can also result in potentially toxic substances, such as biogenic amines (BAs), which are derived from the proteolytic activity of starter or non-starter lactic acid bacteria or contaminating microorganisms (MOs), through the decarboxylation of free amino acids [12]. Among the BAs that can be present in ripened cheeses, tyramine and histamine are the main ones that can potentially affect human health and cause food intoxication when consumed in high quantities. The maximum limit for ingestion will vary according to the food source and type of BA, which may result in allergies, nausea, headache, hypotension, or hypertension [13]. Therefore, it is crucial to investigate these components in ripened cheeses, as the production and concentration of BA can vary depending on the type of cheese and the MOs in the product [12].

A new perspective on the presence of MOs in food has been designed to hypothesize that some daily exposure to harmless MOs—not harmful or commensal microbes—may be beneficial [14]. The rationale for this claim is that harmless live microbes from daily food intake can “bond” with the mucosal surfaces of the digestive tract, tune the immune system, strengthen gut function, and enhance the ability of the human symbiont to mitigate susceptibility to the development of chronic diseases [14].

Studies on autochthonous LAB from dairy products have demonstrated their ability to inhibit pathogenic and spoilage MOs through the production of organic acids and bacteriocins, as well as competitive exclusion, which showcases their probiotic potential and ensures the microbiological safety of the food without compromising the unique characteristics of artisanal cheeses [8,10,15]. Autochthonous strains from goat milk, such as *Lactiplantibacillus plantarum* CNPC003 and *Limosilactobacillus mucosae* CNPC007, have previously demonstrated functional and technological potential, in its isolated form [16,17] and when added to dairy matrices [18,19,20], including fresh and 60-day-aged goat coalho cheese [2,21]. However, no studies have evaluated the influence of *Lactiplantibacillus plantarum* CNPC003 and *Limosilactobacillus mucosae* CNPC007 on the microbiota composition of medium-ripened artisanal goat cheese.

Considering the critical role of microbiota in determining cheese quality, both traditional and molecular methods have been used to track microbial species variations throughout the production and ripening processes [8]. Although traditional microbiological techniques are widely used, they are limited in their ability to accurately assess the entire microbial community, particularly non-culturable and low-abundance cells [22]. DNA-based culture-independent methodologies have become increasingly popular over the past decade due to their effectiveness in providing a more accurate and practical description of microbial communities, regardless of cell viability [9]. However, since DNA-based methods can also detect DNA from dead bacterial cells, they may not offer a completely accurate representation of the viable microbial load at a given timepoint. Therefore, combining DNA-based techniques with culture-dependent microbiological methods can provide a more comprehensive and reliable overview of the microbial community.

Therefore, this study aimed to evaluate the effects of the autochthonous lactic acid bacteria *Lactiplantibacillus plantarum* CNPC003 and *Limosilactobacillus mucosae* CNPC007 on the microbiological safety, microbiota composition (through 16S rRNA metataxonomic sequencing), and biogenic amine production in medium-ripened artisanal goat coalho cheese made from raw or pasteurized milk, during 60 days of ripening at 10 °C. This work stands out for combining traditional microbiological techniques with high-resolution molecular tools to assess microbial dynamics in a cheese matrix with cultural value traditionally made with raw milk. Furthermore, it introduces a promising biotechnological alternative to milk pasteurization by incorporating selected native strains capable of promoting microbial safety while maintaining the artisanal nature of production. These findings may guide future strategies for the improvement of artisanal dairy production, particularly in small-scale settings, by contributing to safer, higher-quality cheeses without compromising their authenticity.

## 2. Materials and Methods

### 2.1. Materials

The milk utilized in this study was sourced from crossbred “Parda Sertaneja” goats residing at Fazenda Carnaúba, located in Taperoá, Paraíba, Brazil. Lyophilized cultures of *Limosilactobacillus mucosae* CNPC007 (Genetic Heritage: BRMCTAA180) and *Lactiplantibacillus plantarum* CNPC003 (formerly *Lactobacillus plantarum* B12, Genetic Heritage: BRMCTAA179) were obtained from the “Collection of Microorganisms of Interest for the Food Industry” at Embrapa Goats and Sheep (Sobral, Ceará, Brazil). These cultures were previously isolated from the milk and cheese, respectively, of Anglo-Nubian goats. The two MOs were investigated based on their promising functional and technological properties for food applications, previously identified at the species level through molecular techniques [16,23].

Additional components employed in this study, including liquid rennet (Ha-La, microbial chymosin enzyme from *Aspergillus niger* var. *awamori*, 1:3000/75 IMCU, Chr. Hansen^®^, Valinhos, Brazil), lactic acid (Chr. Hansen^®^), analytical grade calcium chloride (FMaia^®^ Ltd., Cotia, Brazil), and refined iodized salt (Norte Salineira S.A. Ind. e Com., Areia Branca, Brazil), were commercially acquired.

### 2.2. Preparation of Strain Inoculum and Processing of Goat Coalho Cheese

For the initial stage of cheese production, adjunct autochthonous cultures were inoculated and subsequently added to the milk. Two strains, *Lactiplantibacillus plantarum* CNPC003 and *Limosilactobacillus mucosae* CNPC007, were individually and aseptically introduced at 0.2% (*w*/*v*) into a solution of powdered whole goat milk (Caprilat^®^, Governador Valadares, Brazil) that had been reconstituted with sterilized water (0.13%, *w*/*v*) and adjusted to a temperature of 35 ± 0.5 °C [2,18]. This mixture was then incubated at 37 °C for a duration of 24 h. The enumeration of viable LAB cells in the prepared inoculum involved serial dilutions (10^−1^ to 10^−9^) in sterilized 0.1% (*w*/*v*) peptone water (HiMedia, Mumbai, India), followed by plating on de Man, Rogosa, and Sharpe agar (MRS, HiMedia) and incubation at 37 °C for 48 h. The final concentration of viable LAB cells in the inoculum destined for the bulk cheese milk was determined to be greater than 8 log CFU/mL.

Six variations of goat coalho cheese were prepared: RC—raw goat milk cheese without the autochthonous strains; PC—pasteurized goat milk cheese without the autochthonous strains; RCLP—raw goat milk cheese with *Lactiplantibacillus plantarum* CNPC003; PCLP—pasteurized goat milk cheese with *Lactiplantibacillus plantarum* CNPC003; RCLM—raw goat milk cheese with *Limosilactobacillus mucosae* CNPC007; and PCLM—pasteurized goat milk cheese with *Limosilactobacillus mucosae* CNPC007.

**Figure 1:**
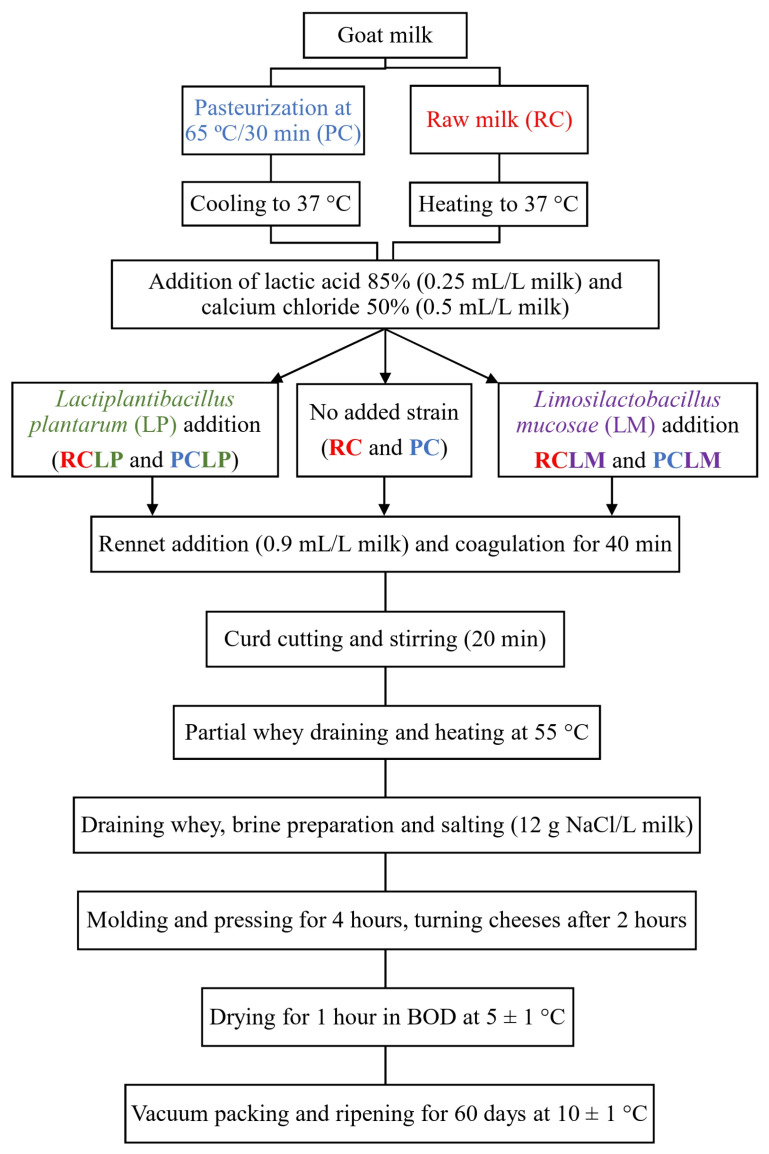
Artisanal goat coalho cheese processing.

The methodology for cheese production was based on a previously established procedure [24], as depicted in Figure 1. Twenty liters of goat milk were used per batch, with half processed raw (for RC, RCLP, and RCLM) and the other half subjected to a low level of pasteurization (65 °C for 30 min, for PC, PCLP, and PCLM). Both raw and pasteurized milk were adjusted to 37 °C before initiating cheese-making. Lactic acid (85%) was added at 0.25 mL/L (diluted 1:10 *v*/*v*), followed by activated *Lactiplantibacillus plantarum* CNPC003 (20 mL/L) for RCLP and PCLP cheeses, and *Limosilactobacillus mucosae* CNPC007 (20 mL/L) for RCLM and PCLM cheeses. Calcium chloride (50%, 0.5 mL/L) and liquid rennet (0.9 mL/L) were then added. Coagulation occurred over 40 min, and after coagulated, the curd was cut using a cheese harp, and then, gently stirred for 20 min. After that, part of the whey that separated from the curd was collected and heated to 55 °C, then added again to the curd and stirred for 5 min. Lastly, most of the whey was drained, and only part of it was collected to produce the brine (12 g NaCl/L of milk), which was reinserted to the cheese curd. After salting, the curd was then molded in perforated rectangular forms, pressed for 4 h, dried at 4 ± 0.5 °C for 1 h in a B.O.D. incubator (Marconi, MA 415, Piracicaba, Brazil), and vacuum-sealed (Tecmaq, TM 150, São Paulo, Brazil).

Each cheese type was produced in two independent batches and analyzed in triplicate at 1, 20, 40, and 60 days of ripening at 10 ± 1 °C for microbiological quality and BA content, with additional metataxonomic analyses conducted at days 1 and 60 of ripening at 10 ± 1 °C. For BA determination and metataxonomic analysis, samples were lyophilized using a bench-top freeze-dryer (model L-101; LIOTOP^®^, São Carlos, Brazil) at −55 ± 2 °C with vacuum pressure below 138 μHg, a lyophilization rate of 1 mm/h, and a drying time of approximately 48 h.

### 2.3. Microbiological Quality of Cheese Samples

The microbiological safety of the cheese samples was evaluated by assessing total and thermotolerant coliforms, coagulase-positive *Staphylococcus* counts, and the presence of *Listeria monocytogenes* and *Salmonella* sp. (per 25 g) according to APHA [25] procedures. The detection and quantification of MOs were performed in triplicate with 25 g of sample, by inoculation, respectively, in the following media: brilliant green bile broth (BGBB) 2%, E.C. broth, mannitol salt agar, Listeria selective agar (Twin Pack) and *Salmonella–Shigella* (SS) agar (HiMedia), followed by incubation at 37 °C for 48 h. Coagulase-positive staphylococci, *Listeria monocytogenes*, and *Salmonella* counts were reported as colony-forming units per gram (CFU/g), while total and thermotolerant coliforms were quantified using the most probable number (MPN) method. The detection thresholds were <1 log CFU/g for coagulase-positive staphylococci and <3 MPN/g for the MPN of thermotolerant and total coliforms.

The viable cell count of potentially probiotic LAB was performed according to APHA [25], dos Santos et al. [26], and de Moraes et al. [2]. Briefly, a cell count was performed after inoculation on MRS agar by the pour plate method and incubation under anaerobiosis (Anaerogen System Anaerogen, Oxoid Ltd., Basingstoke, UK) at 37 °C for 48 h. For cheeses with added *Lactiplantibacillus plantarum*, MRS was supplemented with cysteine (0.5%). The results were expressed as CFU/g of cheese.

### 2.4. Biogenic Amines Determination

Cheese samples were prepared according to Restuccia et al. [27]. Approximately 2.5 g of lyophilized samples, in triplicate, were homogenized with 6 mL of 1 mol/L aqueous hydrochloric acid solution and then supplemented to 10 mL of solution to extract BAs and for the precipitation of proteins in the cheese. The mixture was stirred using a magnetic stirrer (model C-MAG HS 7, IKA, Staufen, Germany) for 30 min and centrifuged (Hermle Z 306, Gosheim, Germany) for 15 min at 3848× *g*. The supernatant was filtered with a 0.45 µm syringe filter (Cobetter, Hangzhou, China), and a 2 mL aliquot of the filtrate was then used for derivatization and subsequent analysis by capillary electrophoresis (CE). For derivatization, 2 mL of the filtered sample was poured into a test tube, and 2 mL of the derivatizer (0.75 g of dansyl chloride per L of acetonitrile) and 0.8 mL of 6 N sodium hydroxide were added to react for 5 min in the absence of light. Then, 1 mL of the supernatant (organic part) was collected, concentrated in a nitrogen stream, and resuspended in 400 µL of acetonitrile. The solution was poured into vials to analyze BA in CE.

Analysis parameters were determined according to Mantoanelli et al. [28]. Initially, capillaries were conditioned by washing with 1.0 mol/L sodium hydroxide solution, followed by washing with ultrapure water and, finally, with running electrolytes, each lasting 30 min. Between each run, conditioning was performed with 0.1 M sodium hydroxide solution, ultrapure water, and running electrolytes for 3 min each. G7100A capillary electrophoresis equipment from Agilent Technologies (Santa Clara, CA, USA) was used, equipped with Diode Array Detection (DAD) (Agilent Technologies), which operates in the range of 190–600 nm, a Peltier temperature control system, and a specific program for data acquisition and processing. The fused silica capillaries used were from Polymicro Technologies, Phoenix, AZ, USA, with dimensions of 75 µm in internal diameter and 48.5 cm in total length, with a detection window of approximately 0.3 cm, opened 40 cm from the detector by removing the polyimide coating from the capillary by heating. Injections were performed through hydrodynamic mode. The results were expressed in mg of BA per kg of cheese, with a detection limit of <0.03 mg/kg.

### 2.5. Metataxonomic Analysis

The cheese samples were analyzed by rRNA V3–V4 region sequencing for the metataxonomic evaluation of the bacterial composition. The outsourced company Neoprospecta Microbiome Technologies (Florianópolis, Brazil) performed sequencing with the Illumina MiSeq platform in paired-end format, using 50 thousand reads per sample. Six replicates of 25 g of each cheese sample were subjected to DNA extraction with a direct analysis procedure using an in-house silica column extraction protocol. A negative extraction control (NEC) was included, which follows the samples in the preparation of the libraries (PCR1 and PCR2). The samples were sequenced in the NextSeq 1000/2000 TM system (Illumina Inc., San Diego, CA, USA) with the NextSeq 1000/2000 P1 600-Cycle Kit.

For bioinformatics analysis, paired-end demultiplexed sequences (paired-end reads) were obtained from the Illumina MiSeq sequencing platform in fastq format and processed by the QIIME2 platform. The sequences were joined, selected by size (>240 bp) according to quality control using the PHRED score (Q > 30), and dereplicated using VSEARCH. Chimeras were verified and removed using UCHIME. Clustering was performed using the de novo method, which had a 99% similarity in obtaining ASVs (amplicon sequence variants). The number of sequences per sample was normalized based on the sample with the lowest number. Taxonomic classification was assigned by the naïve Bayes method in the SILVA database (https://www.arb-silva.de/, accessed on 24 October 2023) with 99% for the V3–V4 region. Diversity and random forest analyses were performed using the MicrobiomeAnalyst platform. Alpha-diversity analyses, namely Shannon, Simpson, and Chao1 analyses, were performed using the Kruskal–Wallis test. Chao1 is a simple estimator of richness, that is, the absolute number of species in a community [29], while Shannon and Simpson are non-parametric (or heterogeneity) indices which cover richness and uniformity simultaneously, as in how evenly distributed they are among these species [30]. A beta-diversity analysis was performed to evaluate the similarity of the microbial profiles among the different treatments of goat coalho cheeses, using the PERMANOVA statistical method using the Bray–Cutis index.

All sequencing data have been deposited in the National Center for Biotechnology Information (NCBI) at the Sequence Read Archive (SRA) under the BioProject accession number PRJNA1247159.

### 2.6. Statistical Analysis

The cheese samples were processed in two independent trials and evaluated in triplicate for microbiological quality assessment and the determination of BAs and in six replicates for metataxonomic analyses. Data related to microbiological analyses and BA determination were subjected to descriptive (mean and standard deviation) and inferential (ANOVA followed by Tukey’s test or Student’s *t*-test) statistics tests to determine significant differences (*p* < 0.05), which were performed using the GraphPad Prism 9.0 software (GraphPad Software Inc., San Diego, CA, USA).

## 3. Results

### 3.1. Microbiological Analysis

**Figure 2:**
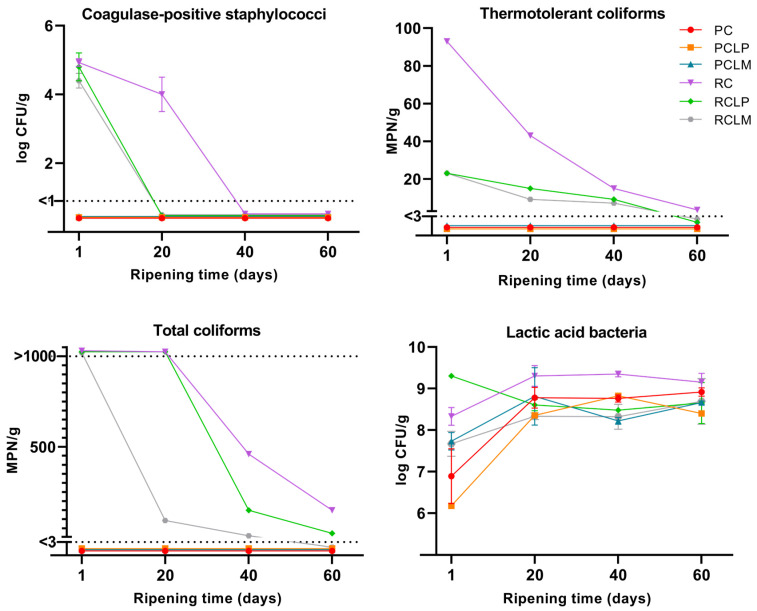
Microbiological counts in goat coalho cheese made from raw or pasteurized milk, with or without autochthonous culture addition, over a 60-day ripening period at 10 °C. PC—pasteurized milk cheese, PCLP—pasteurized milk cheese with added *Lactiplantibacillus plantarum*, PCLM—pasteurized milk cheese with added *Limosilactobacillus mucosae*, RC—raw milk cheese, RCLP—raw milk cheese with added *Lactiplantibacillus plantarum*, and RCLM—raw milk cheese with added *Limosilactobacillus mucosae*.

The microbiological characterization of goat cheese samples made from raw and pasteurized milk was performed throughout the ripening period, and results are presented in Figure 2. The absence of *Listeria monocytogenes* and *Salmonella* sp. in 25 g was observed in all goat cheeses. For raw milk cheese samples, coagulase-positive staphylococci, total coliforms, and thermotolerant coliforms were present at the beginning of the ripening period. For coagulase-positive staphylococci, the RCLP and RCLM samples reached the minimum detection limit (<1 log CFU/g) on the twentieth day of ripening. In contrast, the raw milk sample without strain addition (RC) only reached this limit after 40 days of ripening. A similar behavior was observed for thermotolerant coliforms, where RCLP and RCLM presented the value of 23 MPN/g on the first day of ripening, reaching the minimum detection limit (<3 MPN/g) after 60 days, while RC started with 93 MPN/g, and at the end of ripening, presented the value of 3.6 MPN/g. As for total coliforms, the RCLM sample showed a decrease in growth after 20 days of ripening, reaching counts below the minimum detection limit after 60 days, while RCLP showed a reduction in growth after 40 days, reaching the end ripening with counts of 28 MPN/g. In RC, a higher enumeration of total coliforms was observed, which decreased after 40 days of ripening, reaching 150 MPN/g after 60 days at 10 °C. Thus, it was possible to observe that in samples of raw milk cheese with both *Lactiplantibacillus plantarum* (RCLP) and *Limosilactobacillus mucosae* (RCLM) added, there was a prominent decrease in the growth of the three pathogenic microorganism groups evaluated throughout the ripening period when compared to raw milk cheese without strain addition.

Regarding pasteurized goat milk cheeses, none of the samples presented counts for any pathogenic microorganisms tested, which proves the effectiveness of thermal treatment to control the growth of these pathogens. These results adhere to the requirements and regulations by the country where the research was conducted [31], showing that the goat milk cheeses developed were safe for consumption after 60 days of maturation at 10 ± 0.5 °C.

As for the LAB cell count in goat coalho cheeses, all samples showed a gradual increase throughout the 60-day ripening period. At the beginning of the ripening process, PCLP and PC presented the lowest LAB counts (6.18 and 6.89 log CFU/g, respectively); however, by the end of ripening, all goat cheeses presented counts above 8 log CFU/g.

### 3.2. Biogenic Amines Determination

The content of BAs in goat coalho cheeses was determined throughout the 60-day ripening period at 10 ± 0.5 °C, and the results are presented in Table 1. A significant variability in BA content was observed among the samples evaluated, where PC and PCLM presented a greater variety of amines after 60 days of ripening.

Histamine and tyramine concentrations increased during ripening in all cheeses evaluated (*p* < 0.05), with RCLM and RCLP presenting the highest histamine contents (23.85 ± 0.74 and 17.25 ± 0.63 mg/kg, respectively), and RCLM and RC the highest tyramine contents (1.01 ± 0.01 and 1.00 ± 0.06 mg/kg, respectively) after 60 days (*p* < 0.05). At the end of ripening, the BA spermidine was only present in RC (3.43 ± 0.05 mg/kg), spermine in PC (5.18 ± 1.58 mg/kg), PCLM (4.90 ± 0.30 mg/kg), and RCLM (0.57 ± 0.02 mg/kg), and putrescine only in PCLM (0.16 ± 0.01 mg/kg) and PC (0.11 ± 0.02 mg/kg). It was also observed that the amine cadaverine was present in more significant amounts in PCLM cheeses (21.49 ± 0.88 mg/kg) followed by PC (4.26 ± 0.01 mg/kg) after 60 days of ripening (*p* < 0.05). At the end of the ripening period, RC and PCLP presented the two lowest BA total contents (6.57 mg/kg and 6.96 mg/kg, respectively). In contrast, PCLM presented the highest concentration (32.32 mg/kg).

### 3.3. Metataxonomic Analysis

In parallel with conventional microbiological analysis methods, 16S rRNA gene sequencing of goat cheese samples was also performed to evaluate the bacterial community based on the relative taxa abundance obtained for each treatment and in function of thermal treatment use, with or without autochthonous cultures, and ripening. For this purpose, six replicates were analyzed for each treatment, totaling *n* = 72.

**Figure 3:**
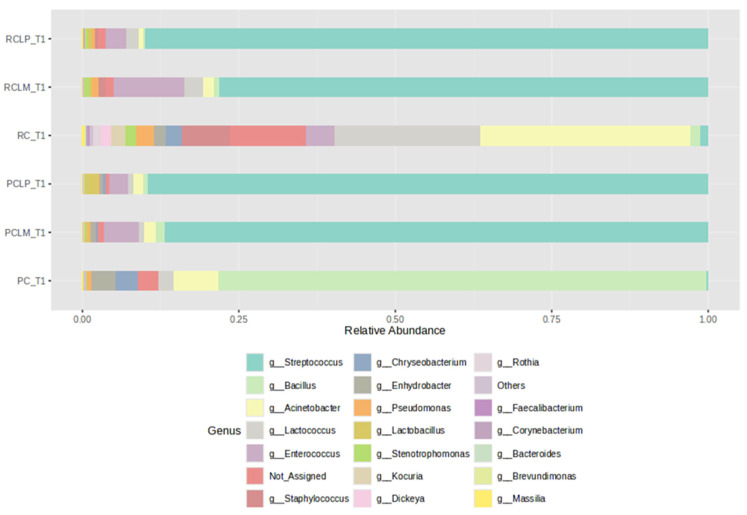
Relative abundance of bacterial genera in goat coalho cheese on the first day of ripening (T1) at 10 °C. PC—pasteurized milk cheese, PCLP—pasteurized milk cheese with added *Lactiplantibacillus plantarum*, PCLM—pasteurized milk cheese with added *Limosilactobacillus mucosae*, RC—raw milk cheese, RCLP—raw milk cheese with added *Lactiplantibacillus plantarum*, and RCLM—raw milk cheese with added *Limosilactobacillus mucosae*.

Figure 3 shows the relative abundances of bacterial genera found in cheese samples on the first day of the ripening period (T1), with a clear difference between samples produced with either raw (RC T1) or pasteurized milk (PC T1). However, after adding autochthonous cultures, the coalho cheese samples presented bacterial profiles with similar percentages of relative abundance among the genera present, despite the use of raw (RCLP T1 and RCLM T1) or pasteurized milk (PCLP T1 and PCLM T1), with a dominance of MOs belonging to the genus *Streptococcus*. The *Staphylococcus* genus, which presents species with pathological potential, was not observed in cheeses obtained from pasteurized milk and represented 7.65%, 0.35%, and 1.2% in the cheeses made from raw milk (RC T1, RCLP T1, and RCLM T1, respectively). Regarding species known as contaminants during cheese production, only *Staphylococcus* was identified.

**Figure 4:**
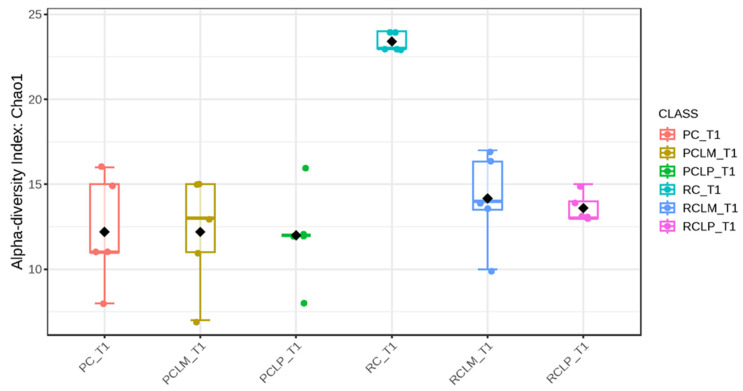
Chao1 index of goat coalho cheese on the first day of ripening (T1) at 10 °C. PC—pasteurized milk cheese, PCLP—pasteurized milk cheese with added *Lactiplantibacillus plantarum*, PCLM—pasteurized milk cheese with added *Limosilactobacillus mucosae*, RC—raw milk cheese, RCLP—raw milk cheese with added *Lactiplantibacillus plantarum*, and RCLM—raw milk cheese with added *Limosilactobacillus mucosae*. *p*-value: 0.014272; [Kruskal–Wallis] statistic: 14.22.

**Figure 5:**
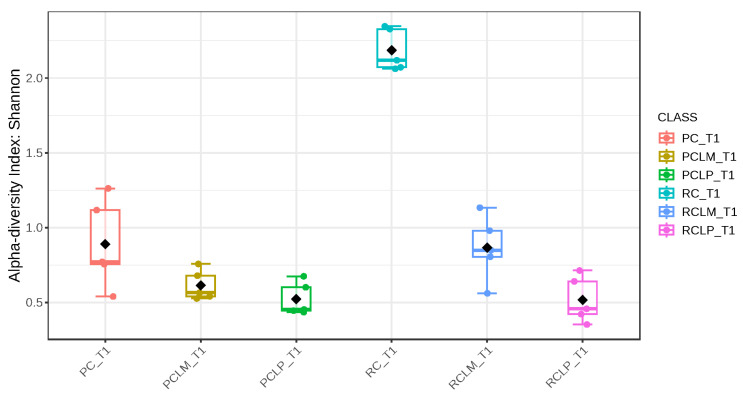
Shannon index of goat coalho cheese on the first day of ripening (T1) at 10 °C. PC—pasteurized milk cheese, PCLP—pasteurized milk cheese with added *Lactiplantibacillus plantarum*, PCLM—pasteurized milk cheese with added *Limosilactobacillus mucosae*, RC—raw milk cheese, RCLP—raw milk cheese with added *Lactiplantibacillus plantarum*, and RCLM—raw milk cheese with added *Limosilactobacillus mucosae*. *p*-value: 0.00106; [Kruskal–Wallis] statistic: 20.381.

**Figure 6:**
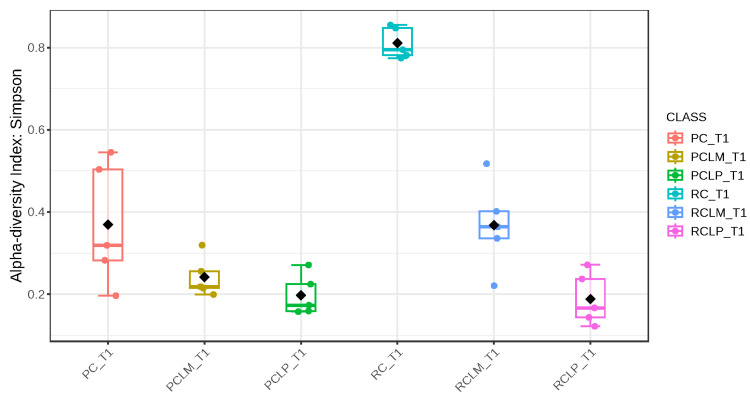
Simpson index of goat coalho cheese on the first day of ripening (T1) at 10 °C. PC—pasteurized milk cheese, PCLP—pasteurized milk cheese with added *Lactiplantibacillus plantarum*, PCLM—pasteurized milk cheese with added *Limosilactobacillus mucosae*, RC—raw milk cheese, RCLP—raw milk cheese with added *Lactiplantibacillus plantarum*, and RCLM—raw milk cheese with added *Limosilactobacillus mucosae*. *p*-value: 0.0011259; [Kruskal–Wallis] statistic: 20.241.

Through alpha-diversity analyses (Chao1, Shannon, and Simpson) of cheese samples on the first day of ripening, it could be observed (Figure 4, Figure 5 and Figure 6, respectively) that the coalho cheese obtained from raw milk and without the addition of autochthonous cultures (RC) presented the greatest bacterial diversity, differing from other treatments.

**Figure 7:**
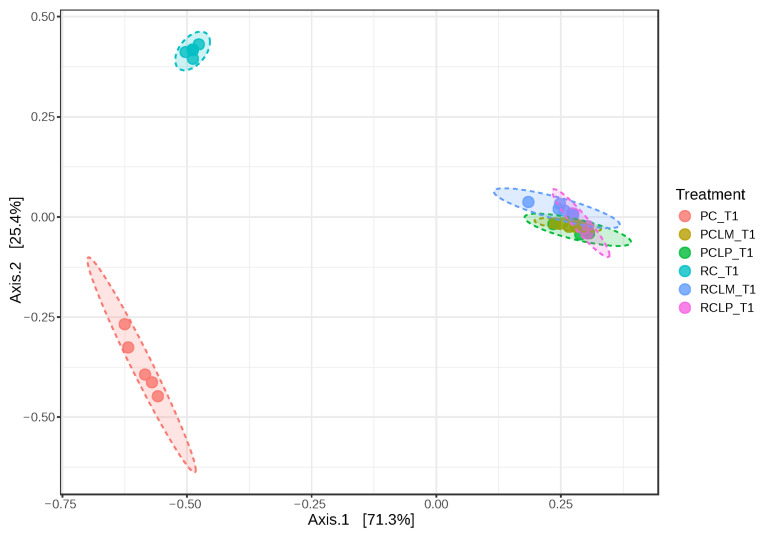
Beta diversity of goat coalho on the first day of ripening (T1) at 10 °C. PC—pasteurized milk cheese, PCLP—pasteurized milk cheese with added *Lactiplantibacillus plantarum*, PCLM—pasteurized milk cheese with added *Limosilactobacillus mucosae*, RC—raw milk cheese, RCLP—raw milk cheese with added *Lactiplantibacillus plantarum*, and RCLM—raw milk cheese with added *Limosilactobacillus mucosae.* [PERMANOVA] F-value: 145.28; R-squared: 0.96802; *p*-value: 0.001.

Beta diversity was also analyzed in goat cheese samples on the first day of ripening (Figure 7). Axis 1 and 2 explained 96.7% of the variation between treatments. Dissimilarity was observed between cheeses obtained from raw (RC T1) and pasteurized (PC T1) milk without adding autochthonous cultures. However, an overlap in similarity was observed between cheeses obtained from raw or pasteurized milk and with added *Lactiplantibacillus plantarum* CNPC003 or *Limosilactobacillus mucosae* CNPC007 strains (RCLP T1, RCLM T1, PCLP T1, and PCLM T1).

**Figure 8:**
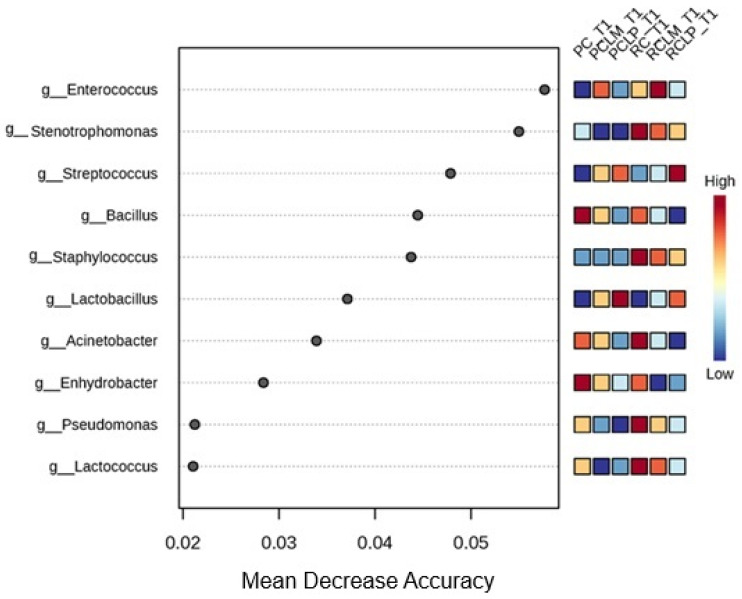
Heat map of bacterial genera in goat coalho cheese on the first day of ripening (T1) at 10 °C. PC—pasteurized milk cheese, PCLP—pasteurized milk cheese with added *Lactiplantibacillus plantarum*, PCLM—pasteurized milk cheese with added *Limosilactobacillus mucosae*, RC—raw milk cheese, RCLP—raw milk cheese with added *Lactiplantibacillus plantarum*, and RCLM—raw milk cheese with added *Limosilactobacillus mucosae*.

Random forest results (Figure 8) reveal differences in the differential abundance of bacterial genera among the various treatments. The predominant genera in cheese produced with raw milk and without culture addition (RC T1) were *Stenotrophomonas*, *Staphylococcus*, *Acinetobacter*, *Pseudomonas,* and *Lactococcus*. With pasteurization (PC T1), a change was observed in microbial composition, with *Bacillus* and *Enhydrobacter* standing out as the genera with the most significant differential abundance.

Autochthonous culture addition also significantly influenced cheese microbiota. At the beginning of ripening, the *Enterococcus* genus showed a higher correlation to raw milk cheese with added *Limosilactobacillus mucosae* CNPC007 (RCLM T1), while in raw milk cheese with added *Lactiplantibacillus plantarum* CNPC003 (RCLP T1), *Streptococcus* was the most abundant. Furthermore, it was observed that PCLP T1 showed a higher differential abundance of *Lactobacillus*.

**Figure 9:**
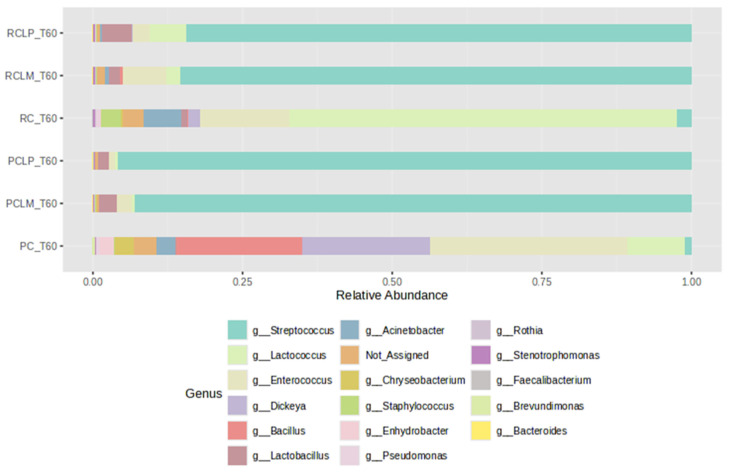
Relative abundance of bacterial genera in goat coalho cheese after 60 days of ripening (T60) at 10 °C. PC—pasteurized milk cheese, PCLP—pasteurized milk cheese with added *Lactiplantibacillus plantarum*, PCLM—pasteurized milk cheese with added *Limosilactobacillus mucosae*, RC—raw milk cheese, RCLP—raw milk cheese with added *Lactiplantibacillus plantarum*, and RCLM—raw milk cheese with added *Limosilactobacillus mucosae*.

For comparison purposes among the different treatments, cheeses produced with pasteurized and raw milk were subjected to ripening for 60 days (T60) and subsequently to bacterial profile analysis and alpha- and beta-diversity indices. As illustrated in Figure 9, after 60 days of ripening, the cheese sample with the microbial profile least like the others was coalho cheese produced with pasteurized milk (PC T60), which presented greater uniformity among a wider variety of bacterial genera. In contrast, a predominance of the genus *Lactococcus* was observed in cheese obtained from raw milk and equally ripened (RC T60).

The addition of autochthonous cultures significantly influenced the bacterial community, promoting similar relative abundance profiles, regardless of thermal treatment, with a predominance of *Streptococcus*. Furthermore, after ripening, the *Staphylococcus* genus was not detected in pasteurized treatments, while in RC T60, RCLP T60, and RCLM T60 cheeses, its presence was 3.18%, 0.11%, and 0.21%, respectively.

**Figure 10:**
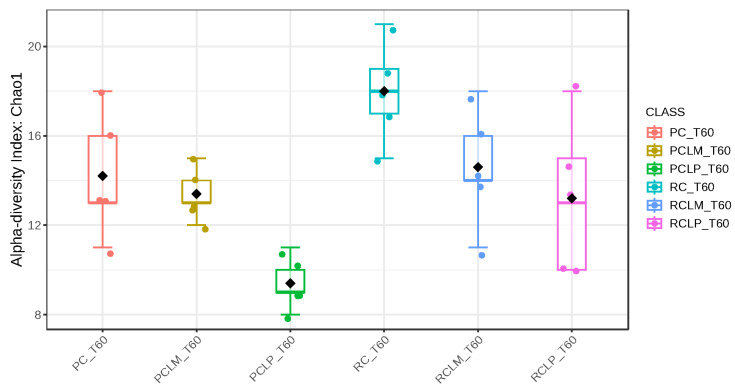
Chao1 index of goat coalho cheese after 60 days of ripening (T60) at 10 °C. PC—pasteurized milk cheese, PCLP—pasteurized milk cheese with added *Lactiplantibacillus plantarum*, PCLM—pasteurized milk cheese with added *Limosilactobacillus mucosae*, RC—raw milk cheese, RCLP—raw milk cheese with added *Lactiplantibacillus plantarum*, and RCLM—raw milk cheese with added *Limosilactobacillus mucosae*. *p*-value: 0.0049899; [Kruskal–Wallis] statistic: 16.754.

**Figure 11:**
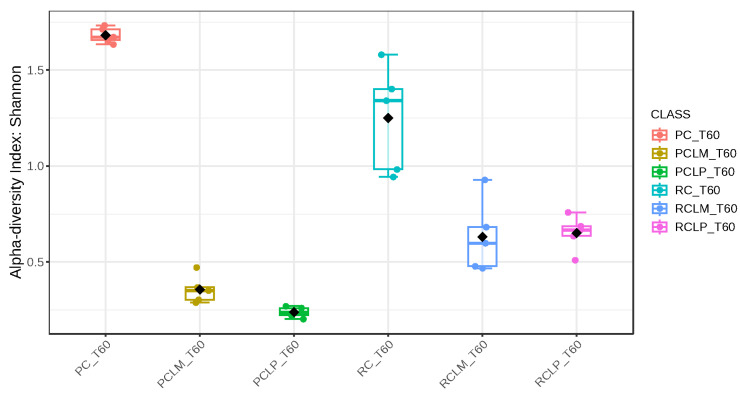
Shannon index of goat coalho cheese after 60 days of ripening (T60) at 10 °C. PC—pasteurized milk cheese, PCLP—pasteurized milk cheese with added *Lactiplantibacillus plantarum*, PCLM—pasteurized milk cheese with added *Limosilactobacillus mucosae*, RC—raw milk cheese, RCLP—raw milk cheese with added *Lactiplantibacillus plantarum*, and RCLM—raw milk cheese with added *Limosilactobacillus mucosae*. *p*-value: 4.7011 × 10^−5^; [Kruskal–Wallis] statistic: 27.431.

**Figure 12:**
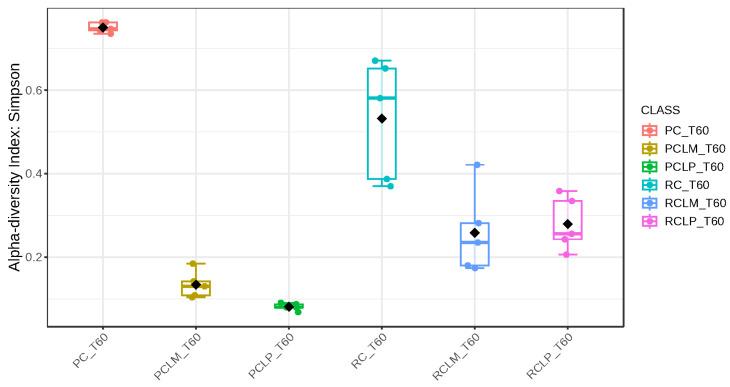
Simpson index of goat coalho cheese after 60 days of ripening (T60) at 10 °C. PC—pasteurized milk cheese, PCLP—pasteurized milk cheese with added *Lactiplantibacillus plantarum*, PCLM—pasteurized milk cheese with added *Limosilactobacillus mucosae*, RC—raw milk cheese, RCLP—raw milk cheese with added *Lactiplantibacillus plantarum*, and RCLM—raw milk cheese with added *Limosilactobacillus mucosae*. *p*-value: 6.5914 × 10^−5^; [Kruskal–Wallis] statistic: 26.677.

Alpha-diversity analyses, based on Chao1, Shannon, and Simpson indices, were performed after 60 days of ripening and are shown in Figure 10, Figure 11 and Figure 12, respectively. According to the Chao1 index (Figure 10), which estimates the absolute number of species in the matrix, raw milk cheese without culture addition (RC T60) maintained the highest microbial diversity. However, the dissimilarity among samples was reduced compared to the start of ripening. According to Shannon and Simpson indices (Figure 11 and Figure 12), which consider both species’ richness and uniformity, it was found that pasteurized milk cheese without culture addition (PC T60) presented the highest bacterial diversity among all samples, which is consistent with the relative abundance results presented in Figure 9.

After the ripening period, according to the Shannon and Simpson indices, it can be observed that the alpha diversity of pasteurized and raw milk cheese samples without culture addition was greater than that of the inoculated samples. Alpha diversity in pasteurized milk cheese (PC T60) also increased significantly compared to the same sample at the start of ripening (PC T1), as seen in Figure 4, Figure 5 and Figure 6. It is thus possible to observe changes in goat cheeses’ bacterial composition resulting from the ripening process, which are linked to the addition or non-addition of autochthonous strains.

**Figure 13:**
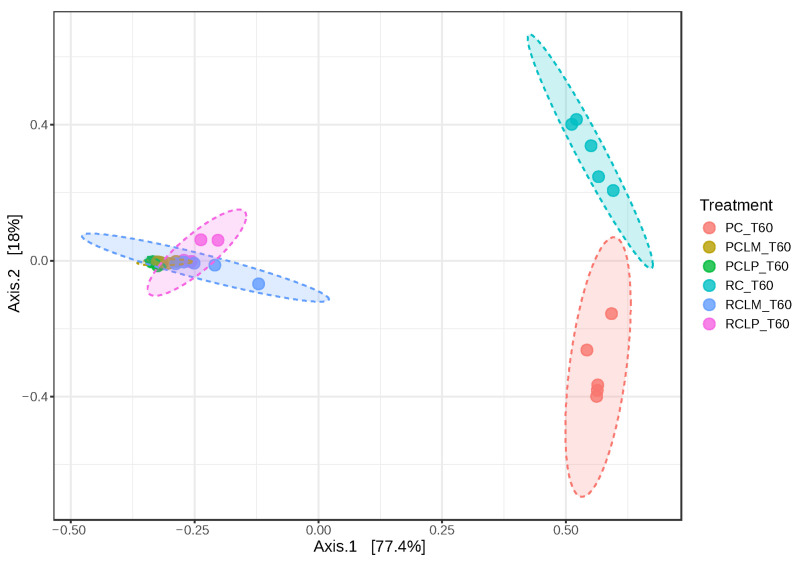
Beta diversity in goat coalho cheese after 60 days of ripening (T60) at 10 °C. PC—pasteurized milk cheese, PCLP—pasteurized milk cheese with added *Lactiplantibacillus plantarum*, PCLM—pasteurized milk cheese with added *Limosilactobacillus mucosae*, RC—raw milk cheese, RCLP—raw milk cheese with added *Lactiplantibacillus plantarum*, and RCLM—raw milk cheese with added *Limosilactobacillus mucosae*. [PERMANOVA] F-value: 72.338; R-squared: 0.93777; *p*-value: 0.001.

Figure 13 illustrates dissimilarities between samples of goat coalho cheese made with pasteurized or raw milk and ripened for 60 days, where axes 1 and 2 explain 95.4% of the observed variations. When comparing the beta-diversity analyses between samples at the start and end of ripening (Figure 7 and Figure 13, respectively), it is noted that samples RC T60 and PC T60 (raw and pasteurized milk cheeses without culture addition) inverted their positions on axis 1 (from negative to positive) and became closer, indicating the significant impact of ripening in the cheese microbiome.

**Figure 14:**
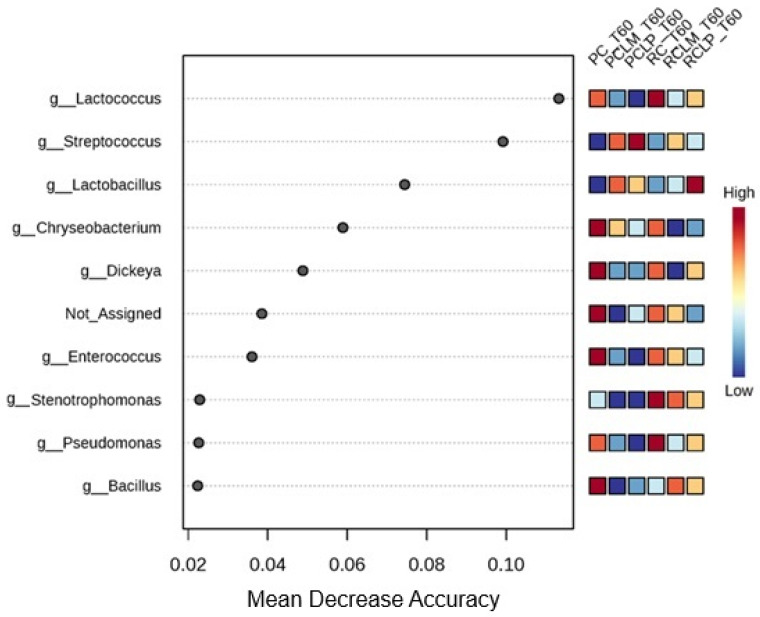
Heat map of bacterial genera in goat coalho cheese after 60 days of ripening (T60) at 10 °C. PC—pasteurized milk cheese, PCLP—pasteurized milk cheese with added *Lactiplantibacillus plantarum*, PCLM—pasteurized milk cheese with added *Limosilactobacillus mucosae*, RC—raw milk cheese, RCLP—raw milk cheese with added *Lactiplantibacillus plantarum*, and RCLM—raw milk cheese with added *Limosilactobacillus mucosae*.

These findings indicate that the addition of both autochthonous cultures, respectively, similarly modulated the microbiota, promoting low dissimilarity between RCLP T60, RCLM T60, PCLP T60, and PCLM T60 treatments, a pattern already observed before ripening (Figure 7). For the ripened samples, a random forest plot was also generated (Figure 14), which revealed differences in the bacterial genera differential abundance. In raw milk-ripened cheese without culture addition (RC T60), there was a predominance of *Lactococcus*, *Stenotrophomonas*, and *Pseudomonas*. In cheeses with added *Lactiplantibacillus plantarum* CNPC003 (RCLP T60 and PCLP T60), a greater differential abundance of *Lactobacillus* (raw milk) and *Streptococcus* (pasteurized milk) was observed, respectively.

## 4. Discussion

Raw milk cheeses are valued for their more intense and complex flavors compared to those produced with pasteurized milk. However, standardizing these products for commercial distribution remains a significant challenge due to the dynamic and variable nature of raw milk microbiota. This variability is influenced by factors such as animal health, diet, environment, and milking practices, all of which impact the microbiological safety of the final product [32,33]. Previous studies have shown that incorporating selected indigenous lactic acid bacteria can enhance the fermentation process, reducing the risks associated with pathogenic microorganisms while preserving the distinctive characteristics of artisanal cheeses [10,32]. In the present study, we used two autochthonous cultures, previously isolated from goat milk and cheese and studied for their probiotic potential [16,18,19], to produce goat coalho cheese from both raw and pasteurized milk, with the goal of improving microbial safety and enhancing consistency in product quality.

In addition to the widely known concept of probiotic MOs, live dietary MOs are an emerging concept, which regards MOs that should be part of the daily diet and thus help the intestinal microbiota become richer and more diverse, as well as promote health benefits [14,34,35]. Several steps are necessary to attest to the functionality of probiotic cultures, culminating in at least one placebo-controlled clinical study [36]. Although the clinical evaluation stage of the *Lactiplantibacillus plantarum* CNPC003 and *Limosilactobacillus mucosae* CNPC007 strains has yet to be performed, published studies prove their safety and functional potential [16,20,21,26].

In the present study, we saw that goat coalho cheese samples presented average counts of total LAB ranging from 8.39 to 9.15 log CFU/g after 60 days of ripening. The recommendation regarding the minimum number of viable probiotic bacterial cells to be ingested to promote health benefits should be 10^6^ to 10^7^ CFU/g of food, with daily consumption of at least 100 g of food containing this probiotic count [37,38,39,40]. However, more recent studies suggest that the recommended dose will depend on the strain used, and each product should be evaluated individually to determine the minimum amount necessary to promote health benefits [41,42,43]. It is essential to clarify that the culture medium for microbiological counting used in this research was not selective for the strains added; however, they characterize the product as a source of live dietary MOs [34,35].

In any case, MO risk must be monitored to ensure food sanitary safety. The coliform bacteria group is widely used as an indicator of milk hygiene, as its presence reflects both the technological level of the production site and the efficiency of refrigeration throughout the production chain [44]. Another pathogen group relevant to milk quality is the coagulase-positive staphylococci bacteria, with *Staphylococcus aureus* as its primary representative. This MO is a contaminant associated with subclinical intramammary infections in ruminants and can compromise the microbiological quality of milk [8]. In cheeses produced with raw milk, although these MO counts were high at the beginning of ripening, a significant reduction was observed over 60 days (Figure 2). This effect was especially notable in cheeses with added autochthonous strains, mostly from the twentieth day of ripening, where RCLP and RCLM started reaching counts closer to pasteurized milk cheeses, suggesting a possible competitive impact of the beneficial microbiota on undesirable MOs.

Similar results were identified in a study by Galdino et al. [45], where the *Lactiplantibacillus plantarum* CNPC003 strain showed an inhibitory effect against *Staphylococcus aureus* ATCC 25923 growth, while *Limosilactobacillus mucosae* CNPC007 inhibited the growth of *Salmonella enterica* serovar Typhimurium ATCC 14028, *S. aureus* ATCC 25923, and *Escherichia coli* ATCC 25922, demonstrating the inhibitory capacity of these autochthonous strains on MOs that indicate sanitary quality. This inhibition can be attributed to the generation of antimicrobial compounds by LAB such as bacteriocins, amino acid metabolites, and organic acids. Naturally present and added LAB in ripened cheeses will produce organic acids, such as lactic acid, through lactose metabolization, causing a pH decrease and consequently allowing these components to penetrate pathogenic MO cell membranes and inhibit their metabolic functions [46]. Previous studies have verified the increase in lactic acid production by *Lactiplantibacillus plantarum* CNPC003 in fresh cheeses, as well as the acidification profile of *Limosilactobacillus mucosae* CNPC007, which could contribute to this inhibition effect [16,20].

A metataxonomic approach was performed to evaluate native goat milk microbiota dynamics and the influence of autochthonous strains on it. Regarding microbiota composition, a predominance of *Streptococcus* was observed for most samples. *Streptococcus* is known to be a thermoduric MO, as it can resist pasteurization. It is noteworthy, however, that this genus was not expressed in great abundance in the sample obtained from raw milk (RC), or even in the cheese produced with pasteurized milk (PC). However, incorporating *Limosilactobacillus mucosae* or *Lactiplantibacillus plantarum,* respectively, into the goat cheese samples favored its greater expression. This is a relevant finding as it highlights the role of microbial ecology in the addition of adjunct cultures in cheese processing. Some species of *Streptococcus*, such as *Streptococcus thermophilus*, benefit cheese quality. Other studies that employed metataxonomic analysis to analyze the microbial composition in different artisanal cheeses produced with raw milk also observed a high relative percentage of the genus *Streptococcus* [47,48].

From the alpha-diversity analyses, it was possible to observe that inoculation of autochthonous strains in raw milk cheeses had an effect like pasteurization in terms of controlling the microbial community and presented better results in maintaining this control and homogeneity of the species by reducing bacterial diversity. Even after the ripening period, inoculated cheeses presented lower MO diversity, which may favor the control and repeatability of desired characteristics in cheese production through the microbial community modulation and reduction of undesirable MO growth during the process. After the ripening period, the *Staphylococcus* genus, which has species with pathogenic potential, presented a relative percentage lower than 0.21% in inoculated cheeses, compared with raw milk cheese (RC). This result shows that inoculation reduced the *Staphylococcus* population, as observed in the plate count method (Figure 2).

Furthermore, it was observed that autochthonous culture inoculation favored beneficial MO growth and promoted greater uniformity of the microbial community. This symmetry, however, does not appear to have been exclusively caused by the inoculated microorganisms since in some cases, such as RCLM T1 cheese, the inoculated strain did not show greater abundance or significant correlation (random forest) with the treatment. However, the presence of this culture appears to have favored *Enterococcus* growth, which suggests complex microbial interactions within the cheese matrix. Furthermore, regardless of milk heat treatment or microorganism inoculation, beta-diversity analysis demonstrated a similarity between the identified species, reinforcing the selection and favoring of specific microbial populations throughout ripening.

The presence of *Enterococcus* in dairy products can be either beneficial or undesirable [32]. On the one hand, this genus contributes to the development of characteristic cheese aromas and flavors through the production of aromatic compounds via lipolysis and proteolysis, in addition to its ability to biosynthesize bacteriocins. However, *Enterococcus* is also recognized as an opportunistic pathogen due to its capacity to produce biogenic BAs and transfer virulence and antibiotic-resistance genes via plasmids. Strains belonging to the genera *Lactobacillus*, *Lactococcus*, and *Streptococcus*, identified during goat coalho cheese ripening, are also commonly associated with BA production [49]. The observed increase in BA concentrations over the 60-day ripening period may be associated with proteolysis by both indigenous and added bacteria, leading to the release of free amino acids, which serve as direct precursors of BAs. Nonetheless, BA levels in goat coalho cheese remained well below the threshold considered hazardous for consumption [12,49].

Among the BAs of most significant concern, histamine stands out, as its concentration tends to increase in ripened cheeses due to the activity of histidine decarboxylase (HDC) enzymes produced by LAB, which can be intensified by temperature and ripening time [50,51]. Some *Streptococcus* spp., such as *S. thermophilus*, have been linked to histamine production, including dairy products, where the HDC enzyme has been shown to resist temperatures close to low pasteurization. This highlights the potential risk of histamine-producing strains in raw milk products [49,52,53].

In this study, cheeses with added autochthonous strains showed a greater relative abundance of *Streptococcus* and significantly higher histamine concentrations after ripening (*p* < 0.05) compared to samples without strain addition. Among these, cheeses made from raw milk (RCLM and RCLP) exhibited the highest histamine concentrations (*p* < 0.05) by the end of ripening. Although no specific regulations exist for histamine limits in all food types, the Commission Regulation [54] sets a 100 mg/kg limit for fish products. In contrast, cheese samples involved in histamine poisoning cases have shown values ranging from 850 to 1870 mg/kg [55]. In this study, even the sample with the highest histamine concentration (RCLM) contained less than 24 mg/kg after 60 days of ripening.

It is important to highlight that those cheeses with added *Lactiplantibacillus plantarum* CNPC003 presented significantly lower concentrations of BAs compared to cheeses with *Limosilactobacillus mucosae* CNPC007, suggesting a positive modulating effect of this strain on the biosynthesis of these substances. However, previous studies with *Lactiplantibacillus plantarum* CNPC003 and *Limosilactobacillus mucosae* CNPC007 have verified the absence of target genes for BA production in both strains [23,26]. Nevertheless, all samples of goat coalho cheese maintained levels of BAs within the recommended standards, including tyramine, whose maximum recommended limit is 6 mg/day. These results indicate that goat coalho cheeses evaluated are safe for consumption, including by individuals using monoamine oxidase inhibitors (MAOIs), who require greater attention regarding the intake of these substances [56].

Overall, these results demonstrate the ability of the autochthonous cultures *Lactiplantibacillus plantarum* CNPC003 and *Limosilactobacillus mucosae* CNPC007 to modulate the microbiota of coalho cheese in a manner like pasteurization, where they may act alone or in combination with the heat treatment. From a technological point of view, these findings are highly relevant as they offer a viable alternative for artisanal producers of goat coalho cheese, enabling microbiological control through biological tools, especially for producers who do not have pasteurizers or work with small volumes of milk. In addition, raw milk can confer differentiated sensory characteristics, often preferred by consumers. Therefore, the findings of this study reinforce the importance of preserving artisanal production techniques, combined with the microbiological safety provided using native cultures of LAB. Furthermore, inoculation with these strains can add functional potential to the cheese, considering its already studied probiotic potential, expanding the possibility of commercial acceptance and guaranteeing the quality and food safety of the product.

## 5. Conclusions

This study demonstrated that the incorporation of the autochthonous strains *Lactiplantibacillus plantarum* CNPC003 and *Limosilactobacillus mucosae* CNPC007 into artisanal goat coalho cheese effectively modulated the microbiota composition, reduced the presence of undesirable microorganisms, and maintained biogenic amine concentrations within safe levels throughout 60 days of ripening at 10 °C. These effects were observed in cheeses made from both raw and pasteurized milk, with the strains promoting a beneficial microbial profile and contributing to improved microbiological safety. The findings provide valuable insights into the potential application of selected autochthonous lactic acid bacteria as a biotechnological strategy for standardizing the quality and safety of medium-ripened cheeses, while preserving their artisanal characteristics, and it is seen that this approach offers an innovative method to enhance microbial control without relying solely on conventional pasteurization. Future research should investigate the sensory characteristics and consumer acceptance of these cheeses and their long-term effects on gut microbiota through in vivo studies. Such studies will be essential to validate the functional properties and commercial applicability of these formulations in the dairy sector.

## Figures and Tables

**Table 1:** Biogenic amine content (mg/kg) in goat coalho cheese with and without adding *Lactiplantibacillus plantarum* CNPC003 and *Limosilactobacillus mucosae* CNPC007 during 60 days of ripening at 10 ± 0.5 °C.

Biogenic Amine (mg/kg)	Ripening Time (Days)	RC	RCLP	RCLM	PC	PCLP	PCLM
Histamine	1	0.09 ± 0.01 ^dC^	0.16 ± 0.01 ^cB^	0.20 ± 0.01 ^cA^	<DL ^bD^	<DL ^cD^	<DL ^dD^
	20	7.18 ± 0.80 ^bD^	20.16 ± 0.44 ^aA^	2.54 ± 0.15 ^bE^	1.67 ± 0.06 ^aE^	9.75 ± 0.79 ^aC^	12.79 ± 0.62 ^aB^
	40	8.85 ± 0.12 ^aD^	17.49 ± 0.53 ^bB^	22.47 ± 0.04 ^aA^	1.21 ± 0.30 ^aE^	10.36 ± 1.11 ^aC^	9.76 ± 1.14 ^bD^
	60	2.14 ± 0.04 ^cD^	17.25 ± 0.63 ^bB^	23.85 ± 0.74 ^aA^	1.48 ± 0.01 ^aD^	6.50 ± 0.04 ^bC^	5.26 ± 0.21 ^cC^
Spermidine	1	<DL ^d^	<DL ^b^	<DL	<DL	<DL	<DL ^b^
	20	183.78 ±15.58 ^aA^	0.59 ± 0.05 ^aC^	<DL ^D^	<DL ^D^	<DL ^D^	<DL ^bD^
	40	44.22 ±2.35 ^bA^	<DL ^bC^	<DL ^C^	<DL ^C^	<DL ^C^	0.27 ± 0.08 ^aB^
	60	3.43 ± 0.05 ^cA^	<DL ^bB^	<DL ^B^	<DL ^B^	<DL ^B^	<DL ^bB^
Spermine	1	<DL	<DL^c^	<DL^c^	<DL^c^	<DL ^b^	<DL ^c^
	20	<DL ^C^	5.48 ± 0.30 ^bB^	6.08 ± 2.49 ^aB^	19.99 ± 1.55 ^aA^	7.03 ± 2.21 ^aB^	2.64 ± 0.02 ^bB^
	40	<DL ^D^	6.86 ± 0.06 ^aB^	<DL ^cD^	3.90 ± 0.67 ^bC^	9.24 ± 0.55 ^aA^	4.10 ± 0.38 ^aC^
	60	<DL ^C^	<DL ^cC^	0.57 ± 0.02 ^bB^	5.18 ± 1.58 ^bA^	<DL ^bC^	4.90 ± 0.30 ^aA^
Putrescine	1	<DL	<DL ^b^	<DL	<DL ^c^	<DL ^b^	<DL ^d^
	20	<DL ^C^	0.11 ± 0.01 ^aA^	<DL ^C^	0.04 ± 0.01 ^bB^	0.07 ± 0.03 ^aAB^	0.07 ± 0.01 ^bAB^
	40	<DL ^B^	<DL ^bB^	<DL ^B^	0.03 ± 0.01 ^bA^	0.04 ± 0.01 ^aA^	0.03 ± 0.01 ^cA^
	60	<DL ^C^	<DL ^bC^	<DL ^C^	0.11 ± 0.02 ^aB^	<DL ^bC^	0.16 ± 0.01 ^aA^
Phenylethylamine	1	<DL	<DL	<DL	<DL	<DL	<DL
	20	<DL	<DL	<DL	<DL	<DL	<DL
	40	<DL	<DL	<DL	<DL	<DL	<DL
	60	<DL	<DL	<DL	<DL	<DL	<DL
Cadaverine	1	<DL ^b^	<DL ^d^	<DL ^d^	<DL ^c^	<DL ^d^	<DL ^d^
	20	0.11 ± 0.01 ^aC^	2.77 ± 0.19 ^bA^	0.77 ± 0.03 ^aB^	0.79 ± 0.08 ^bB^	2.22 ± 0.29 ^bA^	2.14 ± 0.24 ^cA^
	40	<DL ^bD^	13.21 ± 0.13 ^aA^	0.54 ± 0.01 ^bC^	1.64 ± 1.03 ^bC^	15.24 ± 0.64 ^aA^	7.90 ± 2.40 ^bB^
	60	<DL ^bD^	0.15 ± 0.01 ^cC^	0.17 ± 0.01 ^cC^	4.26 ± 0.01 ^aB^	0.06 ± 0.01 ^cC^	21.49 ± 0.88 ^aA^
Tyramine	1	<DL ^d^	<DL ^d^	<DL ^c^	<DL ^b^	<DL ^b^	<DL ^b^
	20	0.12 ± 0.03 ^cCD^	0.31 ± 0.01 ^cB^	<DL ^cE^	0.04 ± 0.03 ^aD^	0.19 ± 0.04 ^aC^	0.43 ± 0.02 ^aA^
	40	0.28 ± 0.02 ^bC^	0.47 ± 0.01 ^bB^	0.67 ± 0.04 ^bA^	<DL ^bD^	0.26 ± 0.04 ^aC^	0.44 ± 0.07 ^aB^
	60	1.00 ± 0.06 ^aA^	0.71 ± 0.01 ^aB^	1.01 ± 0.01 ^aA^	0.09 ± 0.01 ^aE^	0.40 ± 0.07 ^aD^	0.51 ± 0.02 ^aC^
∑ Biogenic amines	1	0.09	0.16	0.20	<DL	<DL	<DL
	20	191.19	29.42	69.55	22.53	19.26	18.07
	40	53.35	38.03	23.68	6.78	35.14	22.50
	60	6.57	18.11	24.60	11.12	6.96	32.32

<DL: below the detection limit (<0.03). PC—pasteurized goat milk cheese; PCLP—pasteurized goat milk cheese with added *Lactiplantibacillus plantarum*; PCLM—pasteurized goat milk cheese with added *Limosilactobacillus mucosae*; RC—raw goat milk cheese; RCLP—raw goat milk cheese with added *Lactiplantibacillus plantarum*; RCLM—raw goat milk cheese with added *Limosilactobacillus mucosae*. a–d: mean ± standard deviation with different lowercase letters within a column differ (*p* < 0.05) among days of ripening, based on Tukey’s test. A–E: mean ± standard deviation with different uppercase letters within a row differ (*p* < 0.05) among cheese formulations, based on Tukey’s test.

## Data Availability

The original contributions presented in this study are included in the article. Further inquiries can be directed to the corresponding author.
